# On the Influence of Large Doses of Quinine in Neuralgia

**Published:** 1851-02

**Authors:** Charles Hogg

**Affiliations:** Finsbury


*On the Influence of Large Doses of Quinine in Neuralgia.* By Charles Hogg, Esq., M.R.C.S., &c., Finsbury.—It is just possible that some of your numerous readers may not be aware of the influence of large doses of quinine in neuralgia. If you think the following worthy of a corner in your valuable periodical, it is at your service.

A case of neuralgia of a very aggravated character occurred in my own famly in the present year, and which yielded, in a most marked manner, to large doses of quinine. My experience leads me to conclude, that in doses of ten grains and upwards, this valuable medicine acts as sedative to the vascular, but highly excitant to the nervous system, as will be seen in the progress of the case. I may here add, that in numerous cases of ague I have found large doses of quinine arrest the progress of that distressing disease. My plan has generally been, to clear out the alimentary canal, by giving two or three grains of calomel, five to eight of compound extract of colocynth, followed by a saline draught; and during the interval of the cold stage, to give the following draught every six hours:—Sulphate of quinine, ten grs.; dilute sulphuric acid, ten minims; distilled water, one ounce: mix: I have seldom had occasion to give more than two doses.

*Neuralgia.*—My patient is twenty-five years of age, and had been for some time back complaining of debility, owing in my opinion, to her suckling and want of rest. She caught a severe cold about Christmas last, which terminated in a violent attack of toothache, resisting every measure I could think of. A few hours' remission followed the extraction of the dens sapientia and free scarification of the gums. The pain now assumed a different character, and was marked by a violent, stabbing, plunging pain, beginning at the left angle of the under jaw, running along the ramus, and upwards in the course of the facial nerve; at other times the pain would be fixed behind the ear, again in the temple, &c., and increased (and even brought on) by the slightest touch of the skin or current of air. An attack would sometimes come on like an electric shock, preceded occasionally by a peculiar twitching of the muscles of the affected side of the face. The loss of sleep and the violence of the pain had disordered the secretions generally. The digestive functions were restored by a pill, composed of two grains of blue-pill, and five of compound extract of colocynth; and a draught, composed of extract of taraxacum, sulphate of magnesia, and tincture of ginger, continued for a few days. I endeavored to procure sleep by Dover's powder, acetate of morphia, hyoscyamus, &c., but witnout success. A slight mitigation of the symptoms were produced by a draught, ordered by my friend Mr. Gay, composed of sulphate of iron, two grains; muriate of morphia, one-sixth of a grain; extract of Canabis Indica, one-twelfth of a grain; it could not be continued, as it seemed to disagree with the child. I had before this prescribed carbonate, sulphate, and citrate of iron, but without avail. I then determined to try the quinine in large doses. She took a draught as follows:—Quinine, ten grains; dilute sulphuric acid, ten minims; distilled water, one ounce. This was followed by distinct remission of the pain, but left a high degree of erethism, a distressing irritability of the acoustic and optic nerve of the affected side, with slight vertigo. Before proceeding further, I obtained the sanction of my friend Dr. Elliotson, who advised the medicine to be continued every sixth hour,—the first dose, ten grains; the next, twelve; and the third, fifteen. After the third dose—that is, the twelve grains, as she had taken two of ten grains—she was completely deaf, particularly on the left side, but had no return of the pain. Generous diet completed the cure. During the last month there has been a slight tendency to a return, which, however, has invariably yielded to two draughts of five grains each. No argument would induce my patient to give up nursing.—*London Lancet.*

